# Supplementary material for: A Single Mutation Increases the Thermostability and Activity of Aspergillus terreus Amine Transaminase
Source: Molecules. 2019 Mar 27;24(7):1194. doi: 10.3390/molecules24071194 (PMC6479498; doi:10.3390/molecules24071194)
Supplement: Supplementary file 1 [file molecules-24-01194-s001.pdf]

Supplementary Materials

A Single Mutation Increases the Thermostability and Activity of *Aspergillus terreus* Amine Transaminase

Wan-Li Zhu [1], Sheng Hu [2], Chang-Jiang Lv [3], Wei-Rui Zhao [2], Hong-peng Wang [3], Jia-Qi Mei [4], Le-He Mei [2,*], Jun Huang [3,*]

[1] College of Pharmaceutical Science, Zhejiang University of Technology, Hangzhou 310014, China; urnotzhuwanli@163.com (W.-l.Z.)

[2] Department of Biological and Pharmaceutical Engineering, Ningbo Institute of Technology, Zhejiang University, Ningbo 315100, China; genegun@zju.edu.cn; zwr166@sohu.com

[3] School of Biological and Chemical Engineering, Zhejiang University of Science and Technology, Hangzhou 310023, China; yangtzelv@zju.edu.cn; wanghongpeng@hotmail.com (H.-p.W.)

[4] Department of Chemical Engineering, University of Utah, Salt Lake City, Utah 84102, United States; meijiaqi123@gmail.com

* Correspondence: meilh@zju.edu.cn.(L.-h.M.); huangjun@zust.edu.cn (J.H.); Tel.: +86-571-879-531-61 (L.-h.M.); +86-571-850-703-96 (J.H.)

Table of Contents

Figure S1. The mutual information network (A) and distance network (B) of AT-ATA provided by MISTIC ....page S-2

Figure S2. A circos representation of the AT-ATA protein family (PF01063) ....page S-3

Figure S3. The alanine scanning of the eight amino acid residues (H76, F115, E117, L118, N181, W184, T274, and T275) ....page S-4

Figure S4. The stabilized mutants screened from the saturation mutation library of the site L118 ....page S-5

Figure S5. The thermal unfolding of wild-type AT-ATA and its mutants were monitored by DSF ....page S-6

Table S1. The primers used for alanine scanning and saturation mutagenesis ....page S-7

Table S2. The stability comparison of wild-type and stabilized single variants by three rational methods ....page S-8

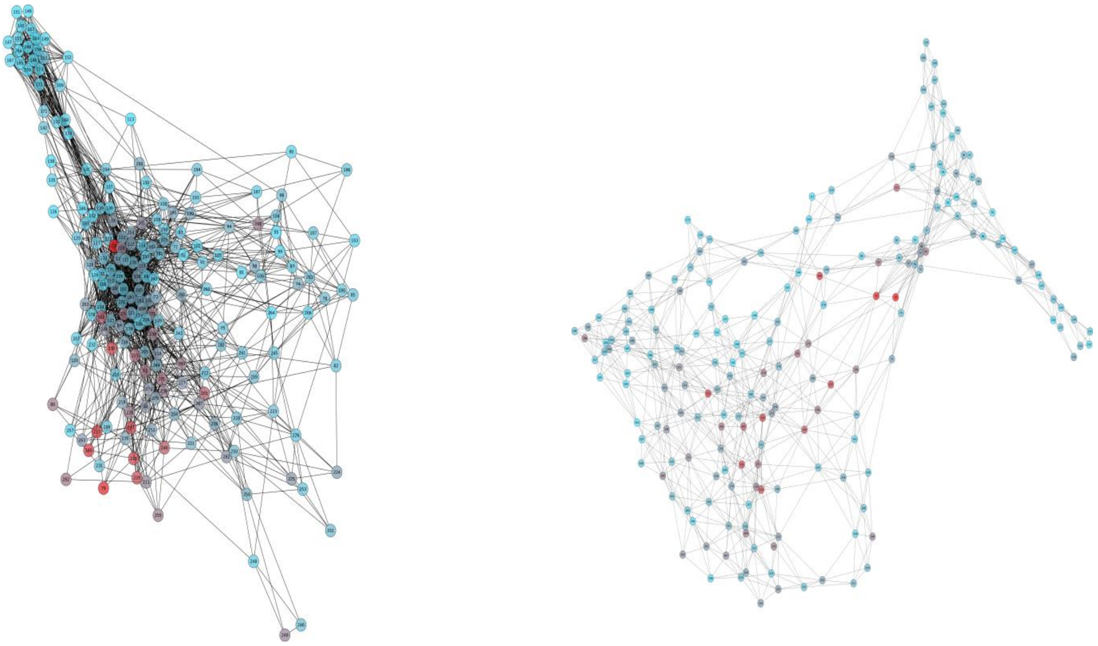

(**A**) (**B**)

Figure S1. The mutual information network (A) and distance network (B) of AT-ATA provided by MISTIC.

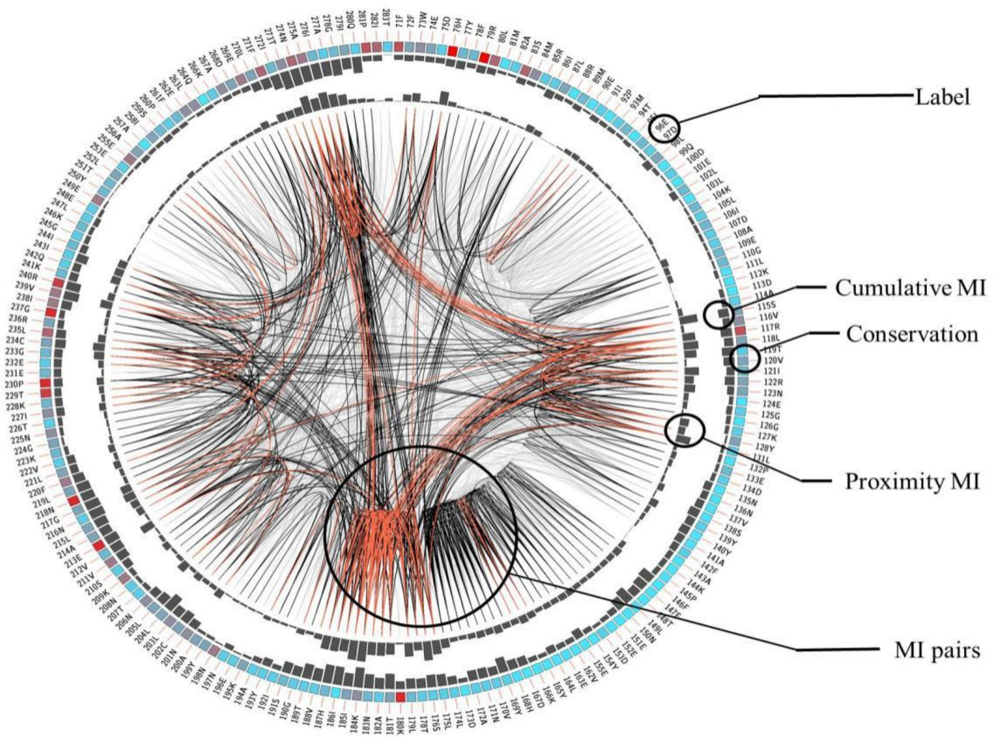

Figure S2. A circos representation of the AT-ATA protein family (PF01063): The labels in the first (outer) circle indicate the amino acid code and the PDB number of the reference sequence. The colored square boxes of the second circle indicate the Kullback–Leibler conservation score (from red to cyan, red: highest; cyan: lowest). The third and fourth circles show the cMI and pMI scores as histograms, facing inward and outward, respectively. The lines in the center of the circle connect pairs of positions with an MI score > 6.5. The red lines represent the top 5%; the black ones are between 70 and 95%, while the gray ones account for the last 70%.

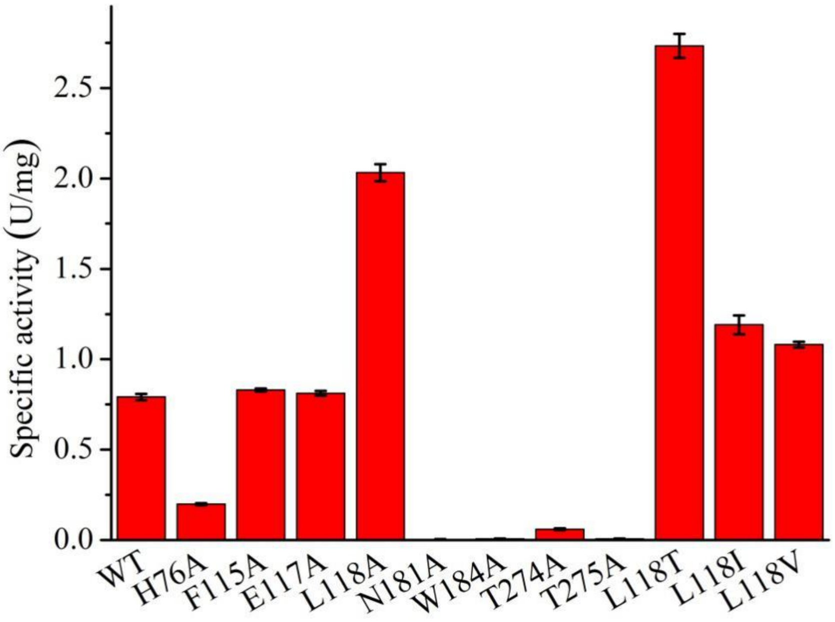

Figure S3. The alanine scanning of the eight amino acid residues (H76, F115, E117, L118, N181, W184, T274, and T275): The enzyme activity of eight single mutants and three mutants (L118T, L118I, and L118V) from saturation mutagenesis were determined and calculated. Each data bar represents the mean ± standard deviation (SD) from three independent measurements.

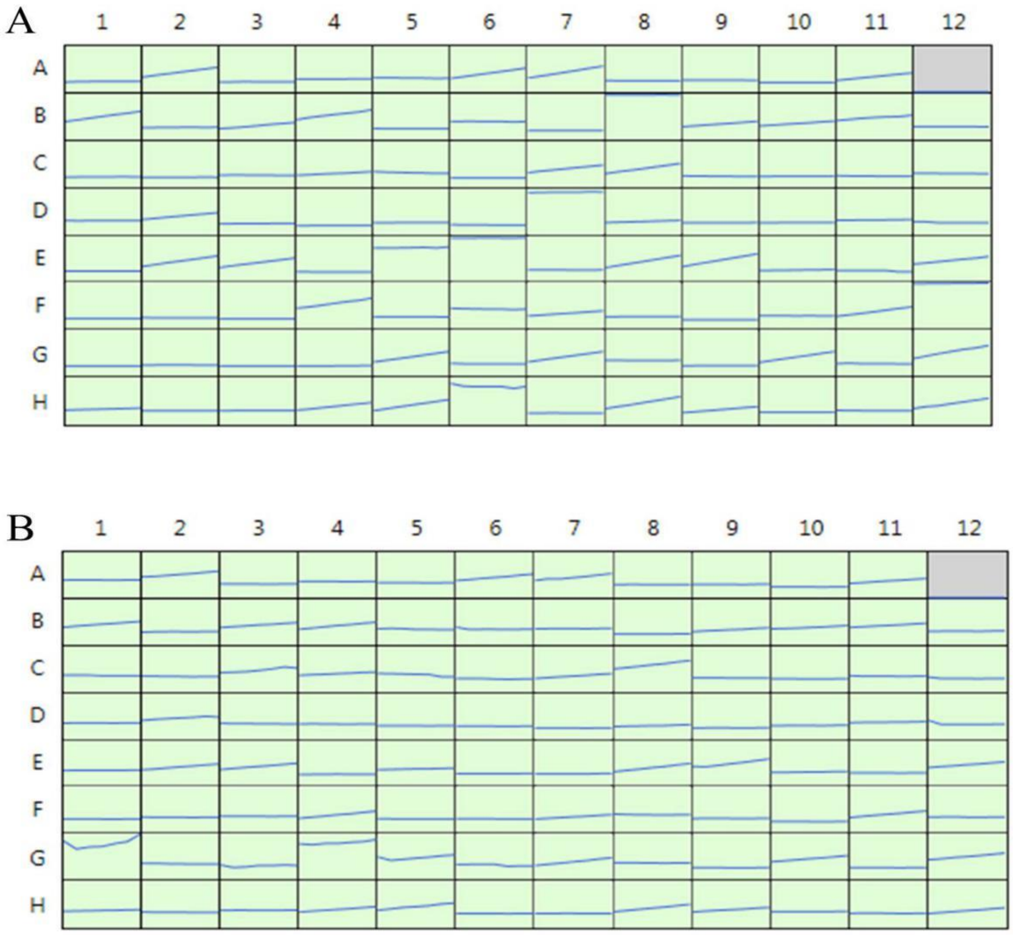

C

	1	2	3	4	5	6	7	8	9	10	11	12
A		32.2				50.9	55.7				53.4	blank
B	52.8		47.1	62.8								
C							77.9	67.8				
D		62.3										
E		69.7	52.9					54.8	51.5			62.1
F				57.6							55.0	
G					63.2		61.2			46.4		41.8
H				55.7	46.4			50.3	58.5			45.1

Residual activity (%)

Figure S4. The stabilized mutants screened from the saturation mutation library of the site L118: (**A**) A 96-well plate of crude enzyme without heat treatment and (**B**) a replica 96-well plate of crude enzyme heated at 50 °C for 10 min. The enzyme activity of each well was determined by measuring the production of acetophenone at pH 8.0 for 3 min. The residual activity of each colony was calculated. (**C**) Those mutant colonies exhibiting nearly similar or even a higher residual activity than wild type (H12) are shown**.**

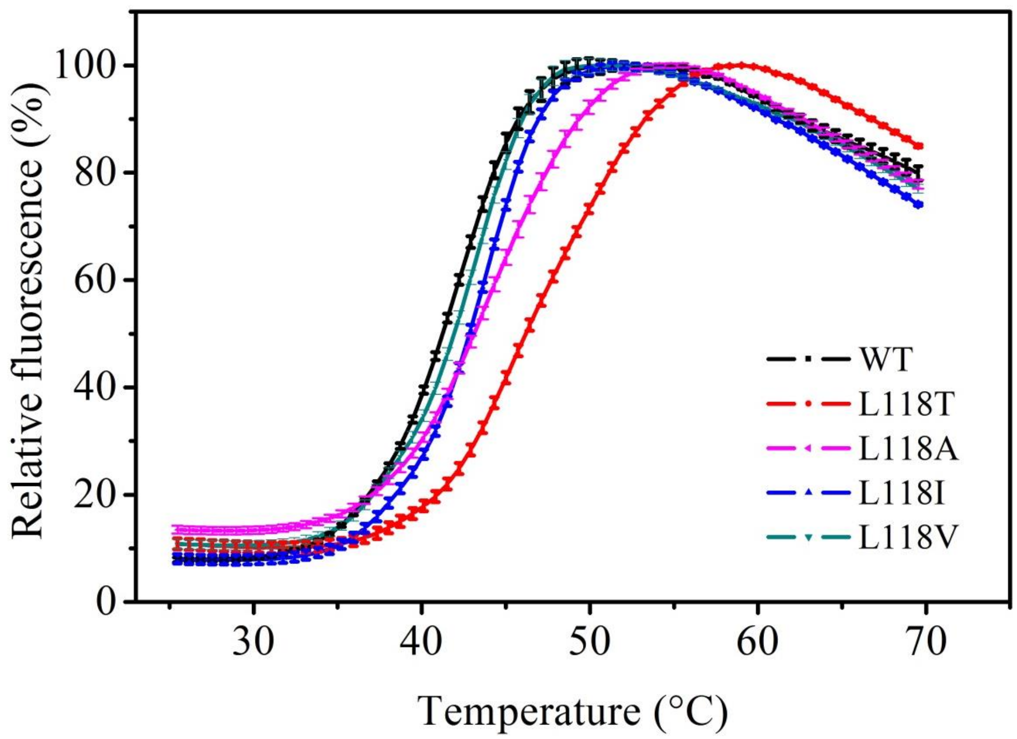

Figure S5. The thermal unfolding of wild-type AT-ATA and its mutants were monitored by DSF.

Table S1. The primers used for alanine scanning and saturation mutagenesis.

Primer name	Primer Sequences (5'–3')
H76A-F	CGTTTAGATGATGCGATTACACGCCTGG
H76A-R	GCTTCCAGGCGTGTAATCGCATCATCTAAAC
F115A-F	GTATTCGGGATGCAGCGGTTGAATTGATAGTC
F115A-R	ACTATCAATTCAACCGCTGCATCCCGAATACCAG
E117A-F	GATGCATTTGTTGCGTTGATAGTCACCCGC
E117A-R	CGGGTGACTATCAACGCAACAAATGCATCC
L118A-F	GCATTTGTTGAAGCGATAGTCACCCG
L118A-R	GCGGGTGACTATCGCTTCAACAAATGCATC
N181A-F	CTATTGATCCGACCGTCAAGGCGCTTCAGTGG
N181A-R	ATCACCCCACTGAAGCGCCTTGACGGTCGGATCAATA
W184A-F	GTCAAGAATCTTCAGGCGGGTGATCTTG
W184A-R	GAACAAGATCACCCGCCTGAAGATTCTTGACG
T274A-F	GACGAGATTTTCATGTGCGCAACGGCGGGTGGC
T274A-R	CATAATGCCACCCGCCGTTGCGCACATGAAAATCT
T275A-F	GATTTTCATGTGCACGGCGGCGGGT
T275A-R	CATAATGCCACCCGCCGCCGTGCACATGAAAAT
L118-F	GCATTTGTTGAANNNATAGTCACCCG
L118-R	GCGGGTGACTATNNNTTCAACAAATGCATC

Table S2. The stability comparison of wild-type and stabilized single variants by three rational methods.

**Mutation name**	**Mutation method**	T_{50}^{10} **(°C)**	ΔT_{50}^{10} **(°C)**	$t_{1/2}$ **(min)**	$t_{1/2}$ **(Fold improvement)**
WT		38.5 ± 0.5	0	6.9 ± 0.6	1
H210N	Consensus	43.1 ± 0.8	4.6	23.1 ± 0.9	3.34
I77L	Consensus	42.8 ± 0.7	4.3	20.1 ± 0.6	2.91
T130M	B-factor and $\Delta\Delta G^{fold}$	42.0 ± 1.2	3.5	15.4 ± 0.4	2.23
Q97E	Consensus	41.7 ± 0.3	3.2	16.5 ± 0.6	2.39
N245D	Consensus	41.4 ± 0.3	2.6	14.8 ± 0.6	2.14
G292D	Consensus	41.3 ± 0.5	2.5	14.8 ± 0.8	2.14
T130F	B-factor and $\Delta\Delta G^{fold}$	40.9 ± 0.7	2.4	13.2 ± 1.6	1.91
E133F	B-factor and $\Delta\Delta G^{fold}$	39.6 ± 0.3	1.1	10.4 ± 0.5	1.51
D134L	B-factor and $\Delta\Delta G^{fold}$	39.2 ± 0.3	0.7	10.1 ± 0.8	1.46
I295V	Consensus	38.7 ± 0.2	0.2	9.3 ± 0.5	1.34
L118T	Mutual information	43.8 ± 0.3	5.3	26.1 ±0 .6	3.78
